# Seed Priming with Magnesium Nitrate Improves Mineral Nutrition and Early Growth of Bambara Groundnut Under Salinity Stress

**DOI:** 10.3390/plants15040626

**Published:** 2026-02-16

**Authors:** Siyabonga Ntshalintshali, Mbukeni Andrew Nkomo, Lungelo Given Buthelezi

**Affiliations:** 1 Faculty of Science, Agriculture, and Engineering, Department of Agriculture, University of Zululand, Main Road, KwaDlangezwa Campus, Empangeni 3886, South Africa; 2 School of Sciences and Agriculture, Westville Campus, University of KwaZulu-Natal, Durban 4000, South Africa

**Keywords:** salinity stress, Bambara groundnut, Mg(NO_3_)_2_ seed priming, ion homeostasis, root Na^+^ sequestration, nitrate signaling

## Abstract

Seed priming studies commonly emphasize growth and physiological responses, yet ionomic regulation and tissue-specific nutrient allocation under salinity stress remain poorly explored, particularly in underutilized crops such as Bambara groundnut (*Vigna subterranea* L.). This study investigated whether Mg(NO_3_)_2_ seed priming, previously shown to enhance salt tolerance, is associated with consistent ionomic patterns in contrasting Bambara groundnut genotypes (BGN-14 and BGN-25). Seeds were primed with 0.03% Mg(NO_3_)_2_ and grown under control or saline conditions (200 mM NaCl) for five weeks. Shoot and root tissues were analyzed for macro- and micronutrient composition using ICP-OES. In BGN-14, salinity caused a marked reduction in shoot fresh weight (−49.5%, *p* < 0.05), whereas Mg(NO_3_)_2_ priming largely mitigated this effect under salinity (−0.4%, *p* > 0.05). Root fresh weight declined numerically under salt stress (−70.1%) and primed + salt conditions (−45.5%), but these changes were not statistically significant. Shoot dry weight increased significantly in primed plants (+83.5%, *p* < 0.05), while salinity reduced SDW (−58.4%); primed + salt plants maintained SDW near control levels (+2.6%). In BGN-25, root biomass was unaffected by treatments, whereas salinity significantly reduced shoot biomass relative to primed plants, with a consistent trend of primed > control > primed + salt > salt. Salinity increased the Na^+^/K^+^ ratio, particularly in roots. In BGN-14, the root Na^+^/K^+^ ratio increased significantly from 1.07 to 4.49 (*p* < 0.05), indicating enhanced Na^+^ accumulation, while shoot ratios increased non-significantly. BGN-25 showed a more moderate increase in shoot ratios and a pronounced rise in root ratios. Principal component analysis revealed distinct nutrient clustering, with Na, Fe, and Al loading strongly under salinity, while Ca, K, Mg, and Cu aligned with improved physiological performance. Although differences between salt and primed + salt treatments were often not statistically significant, several ion ratios and nutrient relationships were numerically enhanced under Mg(NO_3_)_2_ priming. This study builds upon earlier physiological findings (where BGN-14 consistently exhibited a stronger positive response to Mg(NO_3_)_2_ priming, outperforming BGN-25 under salt stress) and provides exploratory, hypothesis-generating evidence that Mg(NO_3_)_2_ priming may contribute to salinity tolerance through coordinated ionomic adjustments, including altered Na^+^ allocation and improved nutrient balance, rather than complete Na^+^ exclusion. These findings highlight the relevance of ionomic responses in understanding stress adaptation in underutilized legume crops.

## 1. Introduction

Bambara groundnut (*Vigna subterranea* (L.) Verdc.) is an indigenous African legume valued for its nutritional quality, resilience to marginal environments, and contribution to food security in semi-arid regions [1,2,3]. The crop produces protein-rich seeds with a balanced amino acid composition and is often cultivated by smallholder farmers under low-input conditions, where it plays a crucial role in sustaining their livelihoods [1,2,3,4]. Despite its agronomic and nutritional importance, Bambara groundnut remains under-researched compared to other grain legumes, particularly in relation to stress physiology and nutrient dynamics [5,6,7].

Salt stress is a major abiotic constraint limiting legume productivity worldwide, affecting one-fifth of the world’s irrigated land (≈60 million ha) and expanding rapidly due to climate change, poor irrigation practices, and soil degradation [8,9,10,11]. In legumes, salt stress disrupts growth and development through osmotic stress, ion toxicity, and nutrient imbalance, ultimately impairing biomass accumulation and yield [12,13,14,15,16,17,18]. Excessive accumulation of sodium (Na^+^) and chloride (Cl^−^) ions interferes with the uptake, transport, and homeostasis of essential nutrients such as potassium (K^+^), calcium (Ca^2+^), magnesium (Mg^2+^), and phosphorus (P), making nutrient regulation a central component of salinity tolerance [19,20].

Seed priming is a practical and cost-effective strategy to enhance stress tolerance in crops. By partially hydrating seeds before sowing, priming improves germination performance, seedling establishment, and subsequent stress resilience [21]. Several priming approaches have been reported, including hydropriming, osmopriming, hormonal priming, and halo-priming. Among these, halo-priming using inorganic salts such as nitrates has shown particular promise in mitigating salinity-induced damage by improving ionic balance, nutrient uptake, and metabolic readiness [22]. Although potassium nitrate (KNO_3_) is widely used for seed priming under salinity stress, the effects of alternative nitrate sources such as magnesium nitrate (Mg(NO_3_)_2_) remain poorly explored. In particular, limited information exists on the role of Mg(NO_3_)_2_ in regulating growth, biomass, and ionomic responses under salt stress. This study addresses this gap by evaluating the effects of Mg(NO_3_)_2_ seed priming on two contrasting Bambara groundnut genotypes (BGN-14 and BGN-25) under salinity. Mg(NO_3_)_2_ may contribute Mg^2+^, which could support chlorophyll formation and enzyme activation, alongside nitrate (NO_3_^−^), which is suggested to act not only as a nitrogen source but also as a signaling molecule during stress adaptation [23,24,25,26,27,28]. This foundational assumption, together with the well-documented effectiveness and predominant use of nitrate-based halo-priming agents such as potassium nitrate (KNO_3_), may have contributed to our decision to explore Mg(NO_3_)_2_ seed priming.

While many priming studies have examined growth, yield, and physiological or biochemical responses, few have explored nutrient allocation, ionomic patterns, or tissue-specific elemental relationships, particularly under salinity stress. This knowledge gap is critical, as nutrient uptake and translocation between roots and shoots highlights adaptive salt tolerance mechanisms. In Bambara groundnut, studies are especially limited, with most neglecting nutrient correlations or ion-omics changes, restricting our understanding of how priming modulates salinity responses at the elemental level.

This current study builds upon our previous work [29], which characterized the physiological and biochemical responses of Bambara groundnut subjected to salt stress and Mg(NO_3_)_2_ seed priming [29]. In our previous report, we noted that BGN-14 appeared to exhibit a more pronounced response to Mg(NO_3_)_2_ priming, leading to improved performance compared with BGN-25 under salt stress [29]. Here, we extend that investigation by focusing specifically on nutrient allocation, translocation, and multivariate ionomic relationships in roots and shoots. Through the integration of tissue-specific nutrient profiling and multivariate analyses, the study seeks to generate hypothesis-driven evidence that Mg(NO_3_)_2_ priming supports salinity tolerance in Bambara groundnut by promoting coordinated ionomic adjustments evident in modified Na^+^ allocation and optimized nutrient balance that may explain the physiological and biochemical responses observed previously [29]. This approach allows us to draw informed conclusions and propose potential mechanisms by which Mg(NO_3_)_2_ priming confers salt tolerance.

## 2. Materials and Methods

### 2.1. Study Site and Experimental Material

The study was conducted at the University of Zululand, KwaDlangezwa Campus, Empangeni, KwaZulu-Natal, South Africa (28°50′59.99″ S, 31°49′59.99″ E). The subtropical site experiences average temperatures of 20–30 °C and annual rainfall of ~908 mm. Bambara groundnut (*Vigna subterranea* L. Verdc.) seeds were obtained from uMvoti Industries CC, KwaZulu-Natal. Two genotypes (BGN-14 and BGN-25) were selected based on seed coat colour, size, and vigour. The growth medium was homogenized sandy loam soil from Port Dunford, air-dried, and sieved (2 mm).

### 2.2. Seed Priming and Salinity Screening

Seeds were firstly primed with four nitrate sources KNO_3_, Mg(NO_3_)_2_, NaNO_3_, and NH_4_NO_3_ at 0, 0.03, 0.2, 0.5, and 1%, and simultaneously exposed to salinity levels of 0 to 400 mM NaCl to identify the optimal priming treatment and salinity threshold. Mg(NO_3_)_2_ at 0.03% was selected as optimal, and 200 mM NaCl as the critical stress level which was used as salt stress throughout the study [29]. Seeds were soaked in the solution for 24 h, air-dried for 15 min, germinated in trays for 10 days, then transplanted into pots following method by Ogbuethi [30].

### 2.3. Experimental Design and Growth Conditions

The main experiment followed a randomized complete block design (RCBD) with four treatments, hydro-primed control, Mg(NO_3_)_2_-primed control, unprimed + 200 mM NaCl, and Mg(NO_3_)_2_-primed + 200 mM NaCl, each replicated six times. Plants were grown in 20 cm pots under natural daylight in a greenhouse. Seedlings were irrigated with water until the two-leaf stage, after which saline treatments received 400 mL of 200 mM NaCl twice weekly, corresponding to an estimated EC of 18–20 dS m^−1^. The pot-trial experiment lasted for 5 weeks to monitor the plants response from seedling and vegetative stages. Each treatment consisted of six replicate pots, with four plants per pot, and each pot was considered one experimental unit. For growth and biomass assessments, measurements were taken from all six replicate pots (*n* = 6). In contrast, due to limited plant material resulting from salinity stress, mineral nutrient analysis was conducted using three biological replicates per treatment (*n* = 3). This approach ensured sufficient material for accurate nutrient quantification while maintaining independent biological replication.

### 2.4. Measurement of Plant Growth and Sample Preparation

Fresh and dry biomass were determined using six replicates per treatment. Fresh weight was measured with a precision analytical balance (ADAM PGW 4502e; maximum capacity 4500 g; accuracy ±0.01 g, Milton Keynes, England). Dry weight was obtained after oven-drying the samples at 65 °C for 72 h.

At harvest, plants were carefully uprooted and gently rinsed with distilled water to remove adhering soil particles. Samples were separated into shoots and roots, which were subsequently oven-dried at 65 °C for 72 h using a laboratory incubator (Labcon Incubator, Model 5016LC, Labcon (Pty) Ltd., Krugersdorp, South Africa). The dried tissues were ground into a fine powder using a laboratory hammer mill (SMC) and sieved through a 0.84 mm mesh. Samples collected from each treatment were treated as biological replicates (*n* = 3).

### 2.5. Target Mineral Element Composition

Plant analyses were conducted at the Cedara Agricultural Research Station, KwaZulu-Natal Department of Agriculture and Rural Development (KZNDARD). Moisture content and ash were determined following modified method by Buthelezi [31].

Elemental analysis was conducted using inductively coupled plasma–optical emission spectrometry (ICP-OES) on an iCAP 7000 system (Thermo Fisher Scientific, Waltham, MA, USA), operated in axial mode. The instrument was set at a radio frequency power of 1350 W, with nebuliser, coolant, and auxiliary gas flow rates of 0.5, 12, and 0.5 L min^−1^, respectively, and a pump speed of 35 rpm. For each genotype, elemental concentrations in both shoots and roots were determined from the mean of three replicate measurements [32]. For sample preparation, approximately 1.0 g of oven-dried shoot or root material was weighed into 100 mL volumetric flasks. Ten millilitres of nitric acid (HNO_3_) was added, and the samples were left to pre-digest overnight. The following day, 8 mL of perchloric acid (HClO_4_) was added, and the contents were gently mixed. Samples were then digested on a heating block, starting at 100 °C and gradually increasing to 260 °C, until the evolution of red nitrogen dioxide (NO_2_) fumes ceased. Digestion was continued until the solution volume was reduced to approximately 3–5 mL, without reaching dryness, and completion was indicated by the solution becoming colourless. After cooling, 10 mL of distilled water was added, and the digest was filtered through Whatman No. 1 filter paper. The resulting filtrate was used for the determination of mineral elements by ICP-OES [33]. The ashes were dissolved in 25 mL of 1 M HCl and diluted four-fold with deionized water prior to the determination of P, K, Ca, Mg, Na, Cu, Zn, Fe, Mn, and Al using ICP-OES [34].

### 2.6. Statistical Analyses

The data collected was subjected to a Two-way Analysis of Variance (ANOVA) utilizing GenStat (23rd edition) software with the significant differences determined by honestly significant difference (HSD) according to Tukey’s test at a (*p* ≤ 0.05) level. Dunnett’s multiple comparisons test was used to compare the treatments (primed, salt, primed + salt) to the control group. Correlation analysis and principal component analysis (PCA) were conducted to evaluate multivariate nutrient patterns across treatments and plant tissues using Jamovi (version 2.6) software. GraphPad Prism (version 8.4.2, 679) software was used to generate the graphical representations of data.

## 3. Results

### 3.1. Mg(NO_3_)_2_ Priming Improves Bambara Groundnut Seedling Growth Under Salt Stress

**Figure 1:**
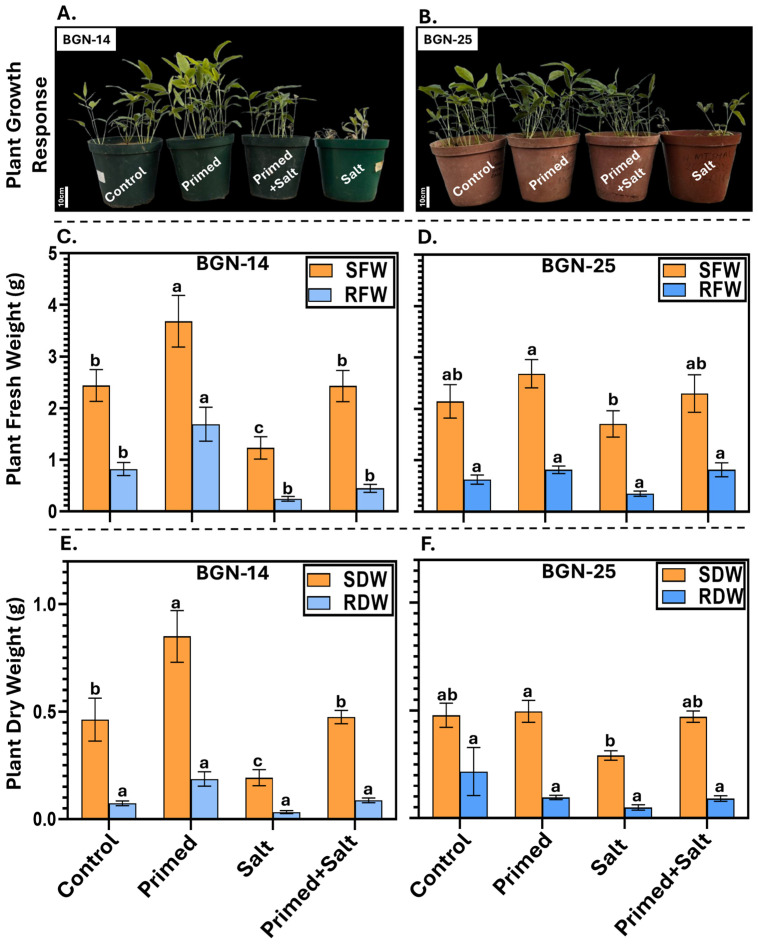
Growth performance of two Bambara groundnut genotypes, BGN-14 and BGN-25, exposed to four treatment conditions: “Control”, “Primed”, “Primed + Salt”, and “Salt”. Representative plants are shown at the top (**A**,**B**). Bar graphs illustrate shoot fresh weight (SFW), root fresh weight (RFW), shoot dry weight (SDW) and root dry weight (RDW) (**C**–**F**). The data are means ± SE (*n* = 6), whereby different letters indicate significant differences among treatments as determined by Tukey’s HSD (*p* < 0.05).

In this study, salt stress had a negative impact on plant growth, with the strongest effects observed in plants exposed to salt only without priming (Figure 1). Reductions in plant height, leaf number, and leaf size were evident under salt stress. These growth reductions were accompanied by changes in biomass allocation (Figure 1A,B). In the BGN-14 genotype, “salt” treatment resulted in significant (*p* < 0.05) reduction in shoot fresh weight (SFW) of approximately −49.52%, whereas only a marginal and non-significant reduction of −0.40% was observed under “primed + salt” treatment when compared to the control (Figure 1C). There were reductions in root fresh weight (RFW) observed for “salt; −70.12%” and “primed + salt; −45.53%” treatments, relative to the control (Figure 1C); however, these reductions where not significant (*p* > 0.05) when compared to the control. Similarly, shoot dry weight (SDW) significantly (*p* < 0.05) increased by +83.45% in “primed” plants, while a reduction of –58.42% and an increase of +2.59% were observed in “salt” and “primed + salt” treatments, respectively, compared with the control (Figure 1E).

In BGN-25 genotype, there were no significant differences observed in RFW and RDW, whilst the SFW and SDW for “salt” treatment was significantly (*p* < 0.05) lower compared to “primed” plants, respectively. The remaining treatments did not differ significantly (*p* > 0.05) (Figure 1).

We observed significant treatment effects on shoot fresh weight (SFW), root fresh weight (RFW), and shoot dry weight (SDW) (Table 1). For instance, for both shoot and root fresh weight, the mean square for treatment was markedly higher than the corresponding error mean square (SFW: MS_T_ = 5.90 vs. MS_E_ = 0.66; RFW: MS_T_ = 1.88 vs. MS_E_ = 0.13), indicating that treatment effects accounted for a substantial proportion of the observed variation. Genotype effects were not significant for any of the measured traits. We detected a significant genotype × treatment interaction for RFW and SDW, while no significant interaction effects were observed for SFW and RDW.

### 3.2. Ionomic Responses of Shoots and Roots to Salinity and Mg(NO_3_)_2_ Priming

There were significant differences (*p* < 0.05) observed in the mineral element composition of Bambara groundnut shoots and roots in genotype BGN-14. For instance, Na content was significantly (*p* < 0.05) higher in the roots of “salt” and “primed + salt” compared to the control. Interestingly, in BGN-25, most of the observed differences among treatments were not significant in either shoots or roots. An exception was Zn concentration, where significant differences were observed across the treatments. Table 2 summarizes the concentrations of major and trace nutrient elements in the shoots and roots of Bambara groundnut genotypes (BGN-14 and BGN-25) under priming, salinity, and combined treatments.

### 3.3. Ion Homeostasis Indicators (Na^+^/K^+^ and Ca^2+^/Na^+^ Ratios)

Salt stress significantly disrupted ionic equilibrium in both Bambara groundnut genotypes, with distinct differences observed between shoots and roots (Table 3). Although the Na^+^/K^+^ ratio increased under salt stress, rising from 0.12 to 0.63 in shoots, under BGN-14, this increase was not significant (*p* > 0.05). The same treatment significantly rose from 1.07 to 4.49 in the roots when compared to the control. Indicating excessive Na^+^ accumulation coupled with reduced K^+^ retention. Although BGN-25 exhibited a more moderate increase in this ratio from 0.24 to 0.32 in shoots and from 2.03 to 4.91 in roots, the changes in shoot ratios for all the treatments were not significant when compared to the control.

The Ca^2+^/Na^+^ ratio declined markedly in BGN-14 under “salt” stress, decreasing from 11.9 to 0.85 in shoots (Table 3). Although there were no significant differences observed in the roots of “primed”, “salt”, and “primed + salt” compared to “control” under BGN-25, this genotype seemed to have maintained relatively lower Ca^2+^/Na^+^ ratios under the “salt” conditions (3.18 to 1.77 in shoots; 0.37 to 0.22 in roots).

## 4. Discussion

### 4.1. Impact of Salinity on Plant Physiology

This study builds on our earlier work [29] to evaluate how Mg(NO_3_)_2_ seed priming influences early growth and shoot–root ionomic responses in Bambara groundnut under salinity stress, providing exploratory insight into coordinated elemental regulation and Na^+^ partitioning. For instance, although “salt” stress significantly reduced shoot fresh and dry biomass compared to “primed” plants, the “primed + salt” treatment partially mitigated these reductions, particularly in BGN-14, which may explain its enhanced performance relative to BGN-25 (Figure 1) [29]. This observation is consistent with numerous studies demonstrating that seed priming enhances stress tolerance and biomass accumulation under adverse conditions [8,22,35,36]. In contrast, the absence of significant differences in root fresh and dry weight between salt-treated and control plants may reflect adaptive root plasticity, whereby root growth is maintained or preferentially allocated under stress to enhance water and ion acquisition, even when shoot growth is compromised. Similar patterns have been reported in other crops, where salinity reduced overall biomass but did not significantly or had less on root mass, especially during early growth stages [37,38].

Notably, the consistent trend of biomass accumulation (primed > control > primed + salt > salt) indicates that Mg(NO_3_)_2_ priming contributed to improved growth performance under salinity. However, the incomplete recovery observed in the “primed + salt” treatment, particularly the lack of significant improvement over “salt-only” plants in SFW and SDW in BGN-25 (Figure 1D,F), suggests genotype-dependent priming efficacy. This limited response supports our earlier findings that BGN-25 is more salt-sensitive, indicating that Mg(NO_3_)_2_ priming was insufficient to fully counteract salinity-induced growth inhibition in this genotype [29].

Similarly, we found that biomass responses were largely driven by treatment effects, with shoot fresh weight showing limited genotypic influence (Table 1). Root fresh weight and shoot dry weight exhibited significant genotype × treatment interactions, indicating genotype-dependent responses to stress conditions. In contrast, root dry weight was less responsive to treatments than fresh weight traits (Table 1), suggesting a stronger influence of treatments on plant water status than on structural biomass. Overall, treatment effects dominated variation across all traits, while genotypic differences were mainly expressed through interaction effects. These findings indicate that priming and salinity primarily modulated biomass and complement our previous observations on physiological and biochemical adjustments [29].

### 4.2. Nutrient-Mediated Regulation of Growth and Stress Tolerance Under Salinity

#### 4.2.1. Macronutrients and Ion Homeostasis Under Salinity Stress

The Na^+^ accumulation in roots under “salt” and “primed + salt” treatments (≈+309% and +267.9% in BGN-14, and +95% under “salt” but −48% under “primed + salt” in BGN-25 relative to control) indicates that salinity-induced toxicity was primarily root-centered (Table 2). Under saline conditions, Na^+^ competes with essential macronutrients such as Ca^2+^, Mg^2+^ and K^+^, which are critical for growth and metabolism [39,40,41], explaining the negative correlations observed between Na and K, Cu and Ca (Figure S1). Genotype-specific Na^+^ regulation was evident, with priming in BGN-14 markedly reducing shoot Na (−79.7%) and slightly lowering root Na (−10%), indicative of enhanced Na exclusion, whereas BGN-25 showed strong root Na reduction (−73.4%) but increased shoot Na (+27.3%) under “primed + salt,” (Table 2) suggesting altered Na compartmentalization or enhanced root-to-shoot translocation. Collectively, these contrasting patterns suggest inherent genetic differences in Na handling strategies under salinity stress.

Salt stress likely interfered with the uptake and internal balance of essential nutrients such as Ca, Mg, P and K, leading to nutrient deficiencies that were visually expressed as growth suppression, chlorosis, necrosis and poor root development under “salt” (Figure 1A,B). Calcium, which is crucial for cell wall stability, membrane integrity, enzyme regulation and signal transduction [42,43], commonly exhibits deficiency symptoms including stunted growth and tissue necrosis under saline conditions [44,45], consistent with the observed salt-stressed phenotype (Figure 1A,B). Similarly, magnesium deficiency, which compromises chlorophyll synthesis, enzyme activation and ATP utilization, leads to reduced chlorophyll content and impaired photosynthetic efficiency [46,47]. In this study, slightly higher Mg levels in “primed + salt” BGN-14 shoots, together with improved chlorophyll content as we observed in our previous study [29], suggest that Mg(NO_3_)_2_ priming partially alleviated salt-induced Mg limitation by supporting early chlorophyll formation and photosynthetic activity, even though genotype-specific differences were evident in BGN-25. Phosphorus deficiency, known to restrict growth through reduced ATP production, nucleic acid synthesis and membrane phospholipid formation [47,48], was also mitigated under “primed” treatments, particularly in BGN-14, where elevated P under “primed + salt” likely enhanced energy availability for stress defense and membrane stabilization, contributing to reduced ROS and lipid peroxidation, as we have observed in our previous study [29]. Potassium imbalance under salinity likely increased osmotic stress and stomatal dysfunction, leading to impaired leaf water status, reduced chlorophyll content, and elevated electrolyte leakage [48,49,50,51,52]. In our previous study we observed that seed-primed plants exhibited significantly higher carotenoid and chlorophyll retention compared with unprimed salt-stressed plants, suggesting that priming might have partially mitigated salinity-induced nutrient and physiological disruptions [29].

#### Ion Homeostasis Indicators (Na^+^/K^+^ and Ca^2+^/Na^+^ Ratios)

While there were no significant differences observed in the shoots of both genotypes in the Na^+^/K^+^ ratios compare to the control, we observed a consistent pattern revealing an increase from shoots to roots, particularly under “salt” and “primed + salt” treatments, with the highest ratio recorded in “salt-only” plants (Table 3). This trend confirms the antagonistic interaction between Na^+^ and K^+^, where excessive Na^+^ limits K^+^ availability and uptake [53,54]. The negative correlations between Na^+^ and K^+^, Ca^2+^ and Cu (Figure S1) further supports the observed antagonistic ion interactions under salinity. The higher Na^+^ concentration we observed in the roots compared with the shoots (see also Figure S2) can be attributed to two main factors: (1st) roots are the primary tissues directly exposed to salinity and therefore accumulate higher Na^+^ and Cl^−^ levels than aerial parts [55] and (2nd) this preferential root accumulation likely represents a protective strategy in Bambara groundnut, whereby excess Na^+^ is sequestered in roots to limit its translocation to shoots and protect photosynthetically active tissues from ionic toxicity [55]. Although Mg(NO_3_)_2_ priming partially alleviated Na^+^ accumulation, it did not fully prevent this effect. The pronounced Na^+^ retention in “primed + salt” roots of BGN-14, coupled with limited shoot translocation, is consistent with root-centered Na^+^ sequestration and restricted xylem loading as key salt-tolerance mechanisms reported in legumes and other crops [56,57].

However, despite previously identifying BGN-14 as the salt-tolerant genotype and BGN-25 as salt-sensitive, we observed unexpectedly more favorable Na^+^/K^+^ and Ca^2+^/Na^+^ ratios in BGN-25 than in BGN-14, particularly under the salt and primed + salt treatments, suggesting genotype-specific differences in ion regulation. This pattern might be explained by two possible mechanisms: (1) BGN-25 might have restricted excessive Na^+^ uptake at the root level, thereby showing lower tissue Na^+^ concentrations and consequently lower Na^+^/K^+^ and higher Ca^2+^/Na^+^ ratios [55]; or (2) these ratios may not accurately reflect the true ionic status at the cellular level, as BGN-14 likely accumulated and compartmentalized Na^+^ into vacuoles or root tissues, resulting in higher total Na^+^ values while still maintaining cytosolic ion homeostasis and functional tolerance. BGN-14 might have accumulated greater total Na^+^ in its tissues through efficient sequestration into vacuoles or root storage sites, thereby protecting the cytoplasm and maintaining metabolic activity [58,59,60,61]. Such compartmentalization would elevate bulk Na^+^/K^+^ ratios even though cytosolic K^+^ homeostasis remained stable. On the other hand, the relatively low Na^+^ content we observed in BGN-25 might be associated with reduced uptake linked with impaired growth and transpiration under stress. Our interpretation aligns with the enhanced physiological and biochemical responses of BGN-14, including higher chlorophyll content, relative water status, and lower superoxide levels, confirming that effective Na^+^ sequestration, rather than absolute ionic ratios, justifies its salt tolerance in our previous report [29].

It is crucial to understand that salinity tolerance is a complex, multigenic trait with differential expression across genotypes. Genotypes may differ in growth maintenance, ion homeostasis, osmotic adjustment, or antioxidant capacity, and these traits do not always align within a single genotype. The literature shows that one genotype can perform well in terms of growth metrics but not in ion regulation, while another mutant genotype may exhibit strong ion homeostasis without enhanced growth performance, reflecting distinct adaptive strategies rather than experimental inconsistency [61,62,63]. We further observed a preferential accumulation of Ca^2+^ and K^+^ in the shoots under both “salt-stressed” and “primed + salt” treatments (see clusters II and V under Figure S2), suggesting enhanced ion translocation from roots to shoots. This pattern likely reflects the roles of Ca^2+^ in maintaining membrane integrity and signaling, and K^+^ in osmotic adjustment and stomatal regulation under saline conditions, thereby supporting shoot physiological function despite ionic stress.

#### 4.2.2. Micronutrients and Antioxidant Defense

Among micronutrients, iron (Fe) is essential for chlorophyll biosynthesis, electron transport within the photosystems, and numerous enzymatic functions [64,65]. Manganese (Mn) is vital for the functioning of photosystem II, enzyme activation, and lignin biosynthesis, with deficiency manifesting as chlorosis and structural weakness [65,66,67]. Similarly, copper (Cu) participates in photosynthetic electron transport, lignification, and antioxidant enzyme regulation, while zinc (Zn) is necessary for enzyme activation, protein synthesis, and auxin metabolism [67,68,69]. Zn deficiency typically results in reduced internode elongation, small leaves, and chlorosis [68,69,70,71]. The availability, deficiency, or toxicity of these micronutrients and their complex interactions, whether antagonistic or synergistic likely influenced how each genotype responded to the treatments.

Importantly, Cu, Zn, and Mn are not only vital micronutrients for general metabolism but also serve as cofactors for superoxide dismutase (SOD) enzymes [71,72,73]. SODs constitute the first line of defense against reactive oxygen species (ROS), catalyzing the dismutation of superoxide radicals (O_2_^−^) into hydrogen peroxide and molecular oxygen. Each SOD isoform requires a specific metal cofactor to function optimally; therefore, the relative availability of these micronutrients is critical for efficient ROS detoxification [73,74,75]. Although we noted an increase in Zn, Cu, and Mn in BGN-14 shoots under “primed + salt” which was not statistically significant (*p* > 0.05) compared to “salt” treatment, there were significant compared to the control group (Table 2), also, the observed association in of Zn and Mn in cluster B (Figure S1) aligns with their shared role as SOD cofactors, supporting the enhanced antioxidant capacity previously observed in BGN-14 [29]. Aluminum (Al), on the other hand, is a non-essential element that becomes toxic in acidic conditions, where it inhibits root elongation and nutrient uptake by binding to phosphate and disrupting Ca and Mg availability. Such interactions may partly explain the separation of these nutrient variables in the PCA biplot (Figure S1).

### 4.3. Integrated Perspective

Building on our previous work, we consistently observed enhanced performance of “primed” plants compared with “control”, “primed + salt” and “salt-stressed” treatments across germination, growth, photosynthetic, water status, and stress-response parameters [29]. These physiological and biochemical responses closely complement the observed ionomic profiles, suggesting a coordinated nutrient-mediated tolerance mechanism. We therefore propose a hypothetical framework in which Mg(NO_3_)_2_ seed priming induces a physiological ‘state of readiness’ before stress exposure. Early nutrient loading with Mg^2+^ and NO_3_^−^ during priming likely supports rapid metabolic activation and early photosynthetic establishment in seedlings exposed to salinity [26]. In addition to serving as a nitrogen source, NO_3_^−^ may function as a signaling molecule, shortening the lag phase during germination and enhancing germination uniformity (higher GRI and SVI), which in turn promotes faster root system development and greater soil exploration [26,75,76,77]. The improved root system likely enhances the acquisition of essential nutrients (K, Mg, P, Zn, Mn), which collectively support growth through ATP production, photosynthesis and assimilate transport, while simultaneously strengthening defense via their roles as cofactors for antioxidant enzymes such as SOD [77,78]. Despite the absence of significant differences in root biomass (Figure 1), the consistently higher root mass in primed + salt plants suggests that Mg(NO_3_)_2_ priming may influence root development at the architectural or physiological level rather than through increased biomass accumulation. Seed priming has been reported to enhance early root vigor, lateral root formation, and nutrient uptake efficiency, traits that may improve plant performance under salinity without necessarily significantly increasing total root dry weight [28,37,38,79]. In this context, the observed improvements in nutrient retention and ion homeostasis may reflect more efficient root function rather than greater root size.

**Figure 2:**
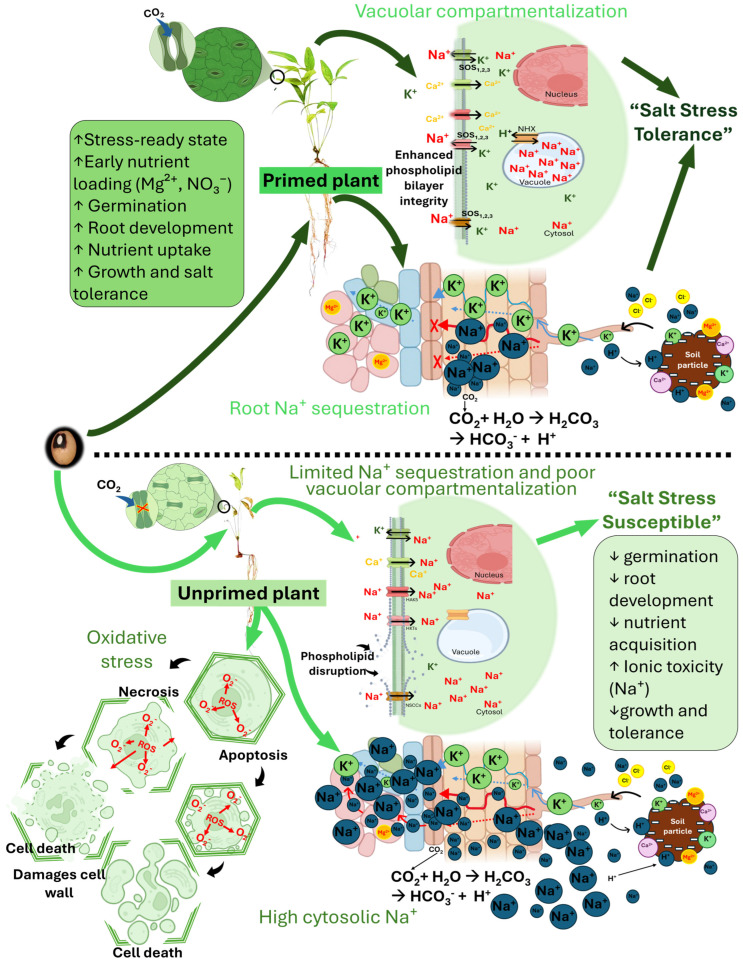
Proposed mechanistic model illustrating the role of Mg(NO_3_)_2_ seed priming in enhancing salt stress tolerance in Bambara groundnut. This figure presents a conceptual framework summarizing the contrasting responses of Mg(NO_3_)_2_-primed and non-primed Bambara groundnut seedlings under salinity stress. Mg(NO_3_)_2_ priming induces a stress-ready state characterized by early magnesium ion (Mg^2+^) and nitrate (NO_3_^−^) nutrient loading, faster and more uniform germination expressed as increased germination percentage (G%), germination rate index (GRI), and seedling vigor index (SVI), enhanced root development, and improved nutrient uptake. Where ↑ = increase, ↓ = decrease. Under salt stress, primed plants exhibit efficient root sodium ion (Na^+^) sequestration and vacuolar compartmentalization mediated by Na^+^/H^+^ antiporters (NHX) and the Salt Overly Sensitive (SOS) pathway, leading to reduced cytosolic Na^+^ toxicity. This is accompanied by improved ion homeostasis, enhanced uptake of essential nutrients including potassium (K^+^), calcium (Ca^2+^), magnesium (Mg^2+^), manganese (Mn), zinc (Zn), and phosphorus (P), and reduced accumulation of reactive oxygen species (ROS). Collectively, these processes sustain photosynthesis, antioxidant defense through superoxide dismutase (SOD), and energy metabolism via adenosine triphosphate (ATP), resulting in improved growth and salt tolerance. In contrast, non-primed seedlings exhibit delayed germination, weak root structures, restricted t uptake of the essential nutrient, excessive cytosolic Na^+^ accumulation, elevated ROS production which can lead to necrosis or apoptosis (damaged cell wall) resulting to cell death, impaired physiological functions, and increased salt susceptibility. Solid arrows indicate the progression from seed priming to physiological outcomes, while cellular schematics illustrate ion transport, compartmentalization, and metabolic consequences under salt stress.

At the same time, priming may have enhanced root-based Na^+^ sequestration and vacuolar compartmentalization, thereby reducing cytosolic ion toxicity. Adequate K availability further supports stomatal regulation, CO_2_ influx, and sustained photosynthesis, contributing to improved biomass accumulation and water status under stress [39,40,41] (Figure 2). In contrast, these coordinated advantages appear compromised in unprimed seeds, where delayed germination, weaker root systems, disrupted ion homeostasis, and reduced antioxidant capacity collectively intensify growth inhibition caused by salt stress. Together, this hypothetical framework provides a mechanistic link between ionomic adjustments and the enhanced physiological and biochemical performance of Mg(NO_3_)_2_-primed Bambara groundnut genotypes under salt stress as depicted in Figure 2.

The proposed mechanistic framework is supported by several studies that investigated Mg(NO_3_)_2_ seed priming, predominantly in cereal crops, where there is broad agreement that Mg(NO_3_)_2_ priming enhances germination, early seedling growth, and vigor, often accompanied by increased enzymatic activity and soluble sugar accumulation [76,80,81,82,83,84,85]. These responses reflect improved metabolic activation during early seedling establishment. However, the underlying mechanisms may slightly differ in legumes due to distinct seed reserve composition and nitrogen metabolism. Furthermore, methodological differences in stress imposition complicate direct comparisons. For example, while Singh et al. [81,82] employed PEG-induced osmotic stress; the present study imposed NaCl-mediated salinity, which integrates both osmotic and ionic stress components. PEG treatments simulate water deficit without introducing toxic ions, whereas NaCl stress directly disrupts ion homeostasis through Na^+^ and Cl^−^ accumulation. As a result, enhanced germination and seedling vigor observed under PEG stress do not necessarily translate to improved Na^+^ exclusion or ionic regulation under true salinity. Our ionomic data therefore extend earlier findings by demonstrating that, under NaCl stress, Mg(NO_3_)_2_ priming likely assists plants to modulate Na^+^ toxicity and nutrient balance rather than making plants completely avoid Na^+^ ions.

## 5. Conclusions and Future Perspectives

In the current study salt stress significantly disrupted nutrient homeostasis in Bambara groundnut, particularly reducing Ca, K, Cu, and Mg while imposing strong Na^+^ toxicity, especially in roots. The genotypes adopted contrasting strategies: BGN-14 accumulated significantly higher root Na^+^, indicating effective sequestration and compartmentalization, which likely protected photosynthetic tissues despite elevated bulk Na^+^/K^+^ ratios, whereas BGN-25 more strongly restricted Na^+^ uptake but showed poorer physiological performance, suggesting that exclusion alone was insufficient for stress tolerance. Mg(NO_3_)_2_ priming partially mitigated these effects, improving K^+^ retention, P availability, and Ca^2+^/Na^+^ balance. It is important to acknowledge that while this study was conducted with methodological rigor, including the use of a conservative HSD post hoc test to minimize experimental error, certain constraints should be acknowledged. Specifically, the ionomic analysis was performed using a limited sample size (*n* = 3) due to restricted plant material availability under salt stress conditions. Although this conservative statistical approach enhances result reliability, it may reduce sensitivity to subtle treatment effects, particularly when variability is high. This likely contributed to the absence of statistically significant differences in some nutrient elements (Table 2), especially in BGN-25, despite the presence of consistent trends and differences in magnitude across treatments. Accordingly, interpretations were cautiously framed to propose plausible mechanisms rather than definitive conclusions. Future studies incorporating larger sample sizes (*n* > 3) would improve statistical resolution by reducing standard errors, thereby enabling more precise mean estimates and stronger inference of nutrient dynamics.

While the present study advances understanding of Mg(NO_3_)_2_ seed priming effects on ionomic regulation in Bambara groundnut under salinity, several other important research gaps remain. First, future studies should incorporate subcellular and transporter-level analyses to distinguish between bulk tissue Na^+^ accumulation and cytosolic ion homeostasis. Quantifying the expression or activity of key Na^+^ and K^+^ transporters (e.g., SOS1, NHX, HKT families) would help confirm whether the observed root-centered Na^+^ accumulation reflects active vacuolar sequestration or restricted xylem loading. Second, given the genotype-specific responses observed between BGN-14 and BGN-25, expanding the analysis to a broader panel of Bambara groundnut genotypes would clarify whether Mg(NO_3_)_2_ priming consistently enhances nutrient redistribution or whether its effectiveness is strongly genotype dependent. This would be particularly valuable for identifying priming-responsive traits that could be targeted in breeding or crop improvement programmes. Third, although our concentration screening identified an optimal Mg(NO_3_)_2_ level, future work should explore dose–response relationships under varying salinity intensities, as priming efficacy may shift under moderate versus severe stress. This would improve recommendations for practical application under heterogeneous field conditions. Fourth, integrating analyses of nutrient dynamics from germination till later developmental stages, such as flowering and yield, would help determine whether priming-induced ionomic adjustments persist beyond early growth or diminish as stress exposure continues. Such studies would bridge the gap between short-term laboratory experiments and long-term field relevance. Finally, combining ionomic profiling with physiological, biochemical, and molecular datasets, including antioxidant enzyme activity, nitrate signaling pathways, and hormonal regulation, would provide a more holistic understanding of how Mg(NO_3_)_2_ priming establishes a stress-resilient phenotype. Collectively, these approaches would strengthen mechanistic inference and support the development of Mg(NO_3_)_2_ priming as a robust, crop-specific strategy for improving salinity tolerance in underutilized legumes such as Bambara groundnut.

## Figures and Tables

**Table 1:** Analysis of variance (ANOVA) showing mean squares (MS) for the effects of genotype and treatment on biomass traits of Bambara groundnut.

Source of Variation	df	MS (SFW)	MS (RFW)	MS (SDW)	MS (RDW)
**Genotype (G)**	1	0.67 ^ns^	0.26 ^ns^	0.04 ^ns^	0.00 ^ns^
**Treatment (T)**	3	5.90 ***	1.88 ***	0.37 ***	0.03 ^ns^
**G × T**	3	1.11 ^ns^	0.86 **	0.12 **	0.03 ^ns^
**Error (E)**	35	0.66	0.13	0.02	0.01

Genotype (G) refers to Bambara groundnut genotypes; Treatment (T) represents the applied priming and salinity conditions; G × T indicates the genotype × treatment interaction. SFW, shoot fresh weight; RFW, root fresh weight; SDW, shoot dry weight; RDW, root dry weight; df, degrees of freedom; Mean squares (MS) are presented. Significance levels are based on F-tests and indicated as: ** *p* ≤ 0.01, *** *p* ≤ 0.001; ^ns^ = not significant. Error represents residual variation.

**Table 2:** Mineral composition (mg kg^−1^ DW) of Bambara groundnut genotypes under control and salt stress in the roots and shoots.

Nutrient Composition (mg kg^−1^ DW)										
Treatment	Ca	Mg	K	Na	Zn	Cu	Mn	Fe	P	Al
BGN-14										
CS	19,035 ± 601	3648 ± 163	12,847 ± 843	1599 ± 140	24 ± 3	13 ± 3	49 ± 3	1502 ± 362	1947 ± 102	453 ± 30
PS	11,106 ± 1280	3120 ± 62	13,960 ± 464	952 ± 127	37 ± 5	12 ± 2	68 ± 7	612 ± 236	3749 ± 314	164 ± 28
SS	13,546 ± 332	3562 ± 377	25,551 ± 2150	16,005 ± 6516	32 ± 3	7 ± 2	37 ± 2	765 ± 200	3292 ± 494	263 ± 41
PSS	19,685 ± 535	4622 ± 199	34,731 ± 1590	11,133 ± 1320	55 ± 12	11 ± 3	39 ± 6	940 ± 354	3654 ± 375	251 ± 52
CR	4554 ± 737	5292 ± 827	5204 ± 1648	5549 ± 957	182 ± 81	6 ± 1	87 ± 11	13,017 ± 2536	1679 ± 141	2317 ± 363
PR	5442 ± 177	9086 ± 1024	9940 ± 1035	7463 ± 1249	165 ± 62	5 ± 1	101 ± 19	9404 ± 1960	1227 ± 38	2340 ± 495
SR	4864 ± 477	3902 ± 688	5058 ± 1100	22,697 ± 2574	67 ± 37	5 ± 1	83 ± 6	10,607 ± 1649	2406 ± 66	1821 ± 286
PSR	3791 ± 581	3279 ± 425	5319 ± 1201	20,417 ± 3389	31 ± 9	5 ± 1	85 ± 7	9854 ± 1404	1943 ± 288	1884 ± 205
*p*-value	<0.001	0.002	<0.001	0.006	0.122	<0.001	<0.001	<0.001	<0.001	0.351
					BGN-25					
CS	14,390 ± 685	3103 ± 143	18,598 ± 719	4531 ± 2325	40 ± 9	10 ± 1	43 ± 3	581 ± 31	3226 ± 109	224 ± 20
PS	19,035 ± 601	3648 ± 163	12,847 ± 843	1599 ± 140	24 ± 2	13 ± 3	49 ± 3	1502 ± 362	1947 ± 102	453 ± 30
SS	19,684 ± 535	4622 ± 199	34,731 ± 1590	11,133 ± 1320	55 ± 12	11 ± 3	39 ± 6	941 ± 354	3654 ± 375	251 ± 52
PSS	13,315 ± 22,569	3643 ± 3897	26,854 ± 15,202	14,172 ± 2821	32 ± 54	10 ± 12	37 ± 73	863 ± 985	3394 ± 2300	236 ± 447
CR	5316 ± 819.3	8502 ± 1409	7066 ± 1267	14,342 ± 469	482 ± 84	13 ± 2	85 ± 1	9484 ± 80	94,835,430 ± 803,414	2141 ± 174
PR	26,733 ± 19,923	35,765 ± 2649	44,156 ± 37,097	31,793 ± 24,787	835 ± 342	29 ± 23	318 ± 211	24,583 ± 15,817	2.46 × 10^8^ ± 1.58 × 10^8^	6939 ± 4667
SR	6103 ± 976	5845 ± 396	5702 ± 460	28,019 ± 2251	131 ± 38	15 ± 1	88 ± 12	8532 ± 1206	85,317,611 ± 12,065,829	1854 ± 264
PSR	6059 ± 4741	6094 ± 797	4163 ± 866	7461 ± 3037	211 ± 42	10 ± 1	78 ± 12	8898 ± 1397	88,980,147 ± 13,971,006	2338 ± 309
*p*-value	0.352	0.279	0.258	0.680	0.017	0.405	0.266	0.467	0.447	0.351

The table summarizes the nutrient content found in the shoots and roots of BGN-14 and BGN-25 across treatments during the seedling stage. Where CS = control shoots, PS = primed shoots, SS = salt shoot, PSS = primed + salt shoot, CR = control roots, PR = primed roots, SR = salt roots, PSR = primed + salt roots, DW = dry weight. The data are means ± SE (*n* = 3).

**Table 3:** Ion homeostasis indicator (Na^+^/K^+^ and Ca^2+^/Na^+^) ratios in shoots and roots of two Bambara groundnut genotypes (BGN-14 and BGN-25) under different treatments.

Genotype	Tissue	Treatment	Na^+^/K^+^	Ca^2+^/Na^+^	Interpretation
BGN-14	Shoot	“Control”	0.12	11.91	Balanced ion status
	Shoot	“Primed”	↓0.07 ^ns^	↓11.67 ^ns^	Slight K^+^ build-up, slight Ca^2+^ decline
	Shoot	“Salt”	↑0.63 ^ns^	↓0.85 ***	Higher Na^+^ build-up, higher Ca^2+^ decline
	Shoot	“Primed + salt”	↑0.32 ^ns^	↓1.77 ***	High Na^+^ build-up and Ca^2+^ decline
	Root	“Control”	1.07	0.82	Normal ionic distribution
	Root	“Primed”	↓0.75 ^ns^	↓0.73 ^ns^	Higher K^+^ build-up, slight Ca^2+^ decline
	Root	“Salt”	↑4.49 ***	↓0.21 ^ns^	Higher Na^+^ accumulation, Ca^2+^ depletion
	Root	“Primed + salt”	↑3.84 ***	↓0.19 ^ns^	High Na^+^ uptake, higher Ca^2+^ depletion
BGN-25	Shoot	“Control”	0.24	3.18	Stable ionic balance
	Shoot	“Primed”	↓0.12 ^ns^	↑11.91 *	Slight K^+^ uptake, Ca^2+^ improvement
	Shoot	“Salt”	↑0.32 ^ns^	↓1.77 ^ns^	Moderate Na^+^ rise, and Ca^2+^ depletion
	Shoot	“Primed + salt”	↑0.53 ^ns^	↓0.94 *	Limited K^+^ restoration, Ca^2+^ depletion
	Root	“Control”	2.03	0.37	Baseline Na^+^ load
	Root	“Primed”	↓0.72 ^ns^	↑0.84 ^ns^	Slight improvement
	Root	“Salt”	↑4.91 ***	↓0.22 ^ns^	Severe Na^+^ accumulation
	Root	“Primed + salt”	↓1.79 ^ns^	↑0.81 ^ns^	Partial mitigation of ionic stress

Ratios were derived from the corresponding mineral concentrations across treatments and are statistically compared to the control group using Dunnett’s multiple comparisons test at a (*p* ≤ 0.05) level where * = *p* < 0.05, *** = *p* < 0.001, ^ns^ = not significant when comparing “primed”, “salt”, “primed + salt” to the control and ↓ = decrease in ratio compared to the control group, ↑ = increase in ratio compared to the control group. However, honestly significant difference (HSD) according to Tukey’s test (*n* = 3) was used to do comparison among all four treatments to each other. Lower Na^+^/K^+^ and higher Ca^2+^/Na^+^ ratios indicate more effective ionic regulation and greater membrane stability under salinity.

## Data Availability

The authors confirm that the data that support the findings of this study are available within the article.

## Supplementary Materials

The following supporting information can be downloaded at https://www.mdpi.com/article/10.3390/plants15040626/s1, Figure S1: Biplot (PCA1 = 66.2%, PCA2 = 21.2%) displaying nutrient loadings and the separation of treatments based on multivariate nutrient variation. Where CS14/CS25 = Control shoot (BGN-14/BGN-25), PS14/ PS25 = Primed shoot (BGN-14/BGN-25), SS14/SS25 = Salt-stressed shoot (BGN-14/BGN-25), PSS14/PSS25 = Primed + salt shoot (BGN-14/BGN-25), CR14/CR25 = Control root (BGN-14/BGN-25), PR14/PR25 = Primed root (BGN-14/BGN-25), SR14/SR25 = Salt-stressed root (BGN-14/BGN-25), PSR14/PSR25 = Primed + salt root (BGN-14/BGN-25). Nutrients include; Ca = calcium, Mg = magnesium, K = potassium, P = phosphorus, Na = sodium, Zn = zinc, Cu = copper, Mn = manganese, Fe = iron, and Al = aluminium; Figure S2: Heatmap illustrating the intensity patterns of nutrient concentrations and the hierarchical clustering of similarity-based grouping of mineral profiles across treatments, tissue types, and genotypes. Where CS14/CS25 = Control shoot (BGN-14/BGN-25), PS14/PS25 = Primed shoot (BGN-14/BGN-25), SS14/SS25 = Salt-stressed shoot (BGN-14/BGN-25), PSS14/PSS25 = Primed + salt shoot (BGN-14/BGN-25), CR14/CR25 = Control root (BGN-14/BGN-25), PR14/PR25 = Primed root (BGN-14/BGN-25), SR14/SR25 = Salt-stressed root (BGN-14/BGN-25), PSR14/PSR25 = Primed + salt root (BGN-14/BGN-25). Nutrients include; Ca = calcium, Mg = magnesium, K = potassium, P = phosphorus, Na = sodium, Zn = zinc, Cu = copper, Mn = manganese, Fe = iron, and Al = aluminium; Table S1: Correlation coefficients among micro and macro-nutrient elements in Bambara ground nut under salt stress; Table S2: Principal component loadings of mineral nutrients and trace elements on the first two principal components (PC1 and PC2).

## Abbreviations

The following abbreviations are used in this manuscript:

AAS	Atomic Absorption Spectroscopy
Al	Aluminium
ANOVA	Analysis of Variance
BGN	Bambara Groundnut
BGN-14	Bambara Groundnut genotype 14
BGN-25	Bambara Groundnut genotype 25
Ca	Calcium
Ca^2+^	Divalent calcium ion
Cl^−^	Chloride ion
Cu	Copper
DW	Dry Weight
EC	Electrical Conductivity
Fe	Iron
FW	Fresh Weight
HCl	Hydrochloric acid
HClO_4_	Perchloric acid
HNO_3_	Nitric acid
HSD	Honestly Significant Difference
HKT	High-Affinity Potassium Transporter
ICP-OES	Inductively Coupled Plasma–Optical Emission Spectrometry
K	Potassium
K^+^	Potassium ion
KNO_3_	Potassium nitrate
Mg	Magnesium
Mg^2+^	Divalent magnesium ion
Mg(NO_3_)_2_	Magnesium nitrate
Mn	Manganese
Na	Sodium
Na^+^	Sodium ion
NaCl	Sodium chloride
NH_4_NO_3_	Ammonium nitrate
NHX	Na^+^/H^+^ Exchanger
NO_2_	Nitrogen dioxide
NO_3_^−^	Nitrate ion
P	Phosphorus
PCA	Principal Component Analysis
RCBD	Randomized Complete Block Design
RFW	Root Fresh Weight
SFW	Shoot Fresh Weight
SOS1	Salt Overly Sensitive 1 transporter
SS	Salt stress
Zn	Zinc
