# Supplementary material for: Seed Priming with Magnesium Nitrate Improves Mineral Nutrition and Early Growth of Bambara Groundnut Under Salinity Stress
Source: Plants (Basel). 2026 Feb 16;15(4):626. doi: 10.3390/plants15040626 (PMC12944371; doi:10.3390/plants15040626)
Supplement: Supplementary file 1 [file plants-15-00626-s001.zip › plants-4112692-supplementary.pdf]

Overall correlation patterns among mineral elements in shoots and roots of Bambara groundnut

The correlation analysis showed clear and meaningful relationships among the nutrients. Magnesium had the strongest positive correlations (values ≥0.6) with almost all other elements, especially Mn, Zn, Al, and P (Table 1S). Calcium (Ca) showed strong and significant positive correlations with potassium (K) and copper (Cu). Potassium and Cu were also significantly correlated (r = 0.567). Sodium (Na) exhibited positive correlations with zinc (Zn), manganese (Mn), iron (Fe), phosphorus (P), and aluminium (Al). Copper was significantly correlated with both Mn and P (r = 0.705 and 0.823, respectively). In addition, Mn and Fe showed significant positive associations with P and Al (Table 1S).

Table 1S. Correlation coefficients among micro and macro-nutrient elements in Bambara ground nut under salt stress.

Var.	Ca	Mg	K	Na	Zn	Cu	Mn	Fe	P
Mg	0.441	—							
K	**0.851*****	**0.501***	—						
Na	-0.018	**0.553***	0.226	—					
Zn	0.218	**0.919*****	0.295	**0.511***	—				
Cu	**0.692****	**0.793*****	**0.567***	0.381	**0.714****	—			
Mn	0.263	**0.965*****	0.312	**0.594***	**0.892*****	**0.705****	—		
Fe	-0.118	**0.795*****	-0.024	**0.626****	**0.825*****	0.407	**0.892*****	—	
P	0.266	**0.893*****	0.295	**0.612***	**0.927*****	**0.823*****	**0.873*****	**0.766*****	—
Al	0.067	**0.906*****	0.140	**0.613***	**0.899*****	**0.570***	**0.960*****	**0.971*****	**0.867*****

Note: * $p < 0.05$, ** $p < 0.01$, *** $p < 0.001$. Correlation coefficients ≥ 0.5 were considered to be significant and highlighted in bold. Var.= Variables, Ca =calcium, Mg= magnesium, K= potassium, P= phosphorus, Na= sodium, Zn= zinc, Cu= copper, Mn= manganese, Fe= iron, Al= aluminium.

Principal component and cluster analyses

The principal component (PC1), which accounted for 66.2% of the variation, was strongly influenced by several nutrients especially Mg, Mn, P, Zn, Al, Fe, Cu, and Na; all of which showed high positive loadings (Table 2S). These nutrients were shown to collectively increase or decrease, forming a common trend across genotypes and treatments. PC2, which explained an additional 21.2%, was shaped mainly by Ca and K, with Cu contributing to a lesser extent (Table 2S). In the biplot, PC1, which explains most of the variation, separates the nutrient elements into three major clusters: A, B and C (Figure 1S). Cluster C, positioned on the lower left of the biplot, was driven largely by high

loadings of Fe, Al, Na, and cluster B consisting of Mn, Zn, Mg and P. Cluster A, on the upper left, aligned more closely with Ca, K, Mg, and Cu (Figure 1S).

Table 2S. Principal Component Loadings of Mineral Nutrients and Trace Elements on the First Two Principal Components (PC1 and PC2).

Variables	PC1	PC2
Ca	0.357	**0.9094**
Mg	**0.981**	0.0934
K	0.421	**0.8119**
Na	**0.652**	-0.2358
Zn	**0.939**	-0.1040
Cu	**0.803**	0.4456
Mn	**0.966**	-0.1109
Fe	**0.841**	-0.4920
P	**0.946**	-0.0515
Al	**0.932**	-0.3166
Eigenvalue	6.62	2.12
Variability (%)	66.2	21.2
Cumulative (%)	66.2	87.4

The PCA simplified the nutrient data into two main components that together captured 87.4% of the overall variation, showing that most of the nutrient differences across treatments were well explained by these two axes, where values (≥0.6) are deemed significant and highlighted in bold.PC1–2 = Principal component. Values ≥ 0.6 were considered to be significant and are emphasized in bold. Variables; Ca= calcium, Mg= magnesium, K= potassium, P= phosphorus, Na= sodium, Zn= zinc, Cu= copper, Mn= manganese, Fe= iron, Al= aluminium.

The hierarchical clustering heat map (Figure 2S) clearly separated root and shoot tissues into two major clusters (Clusters IV and V). Within each tissue group, “control” and “primed treatments clustered closely, indicating similar nutrient profiles. In contrast, “salt” and “primed + salt” treatments formed distinct clusters, reflecting altered nutrient patterns under salinity. At the sub-cluster level, the “primed + salt” treatment partially overlapped with the “control”, indicating a closer resemblance in elemental composition between these treatments.

The heat map patterns closely matched those observed in the PCA, supporting the multivariate structure identified by the principal component analysis. Cluster I grouped a treatment with higher levels of P, Zn, Al, Fe, Na, and Mn, mainly in root tissues (Clusters III and IV), and were largely associated with salt-stressed roots, in line with their higher factor loadings in the PCA biplot. In contrast, Cluster II was characterized by higher concentrations of Ca, K, Mg, and Cu, predominantly in shoot tissues grouped within Cluster V (Figure 2S).

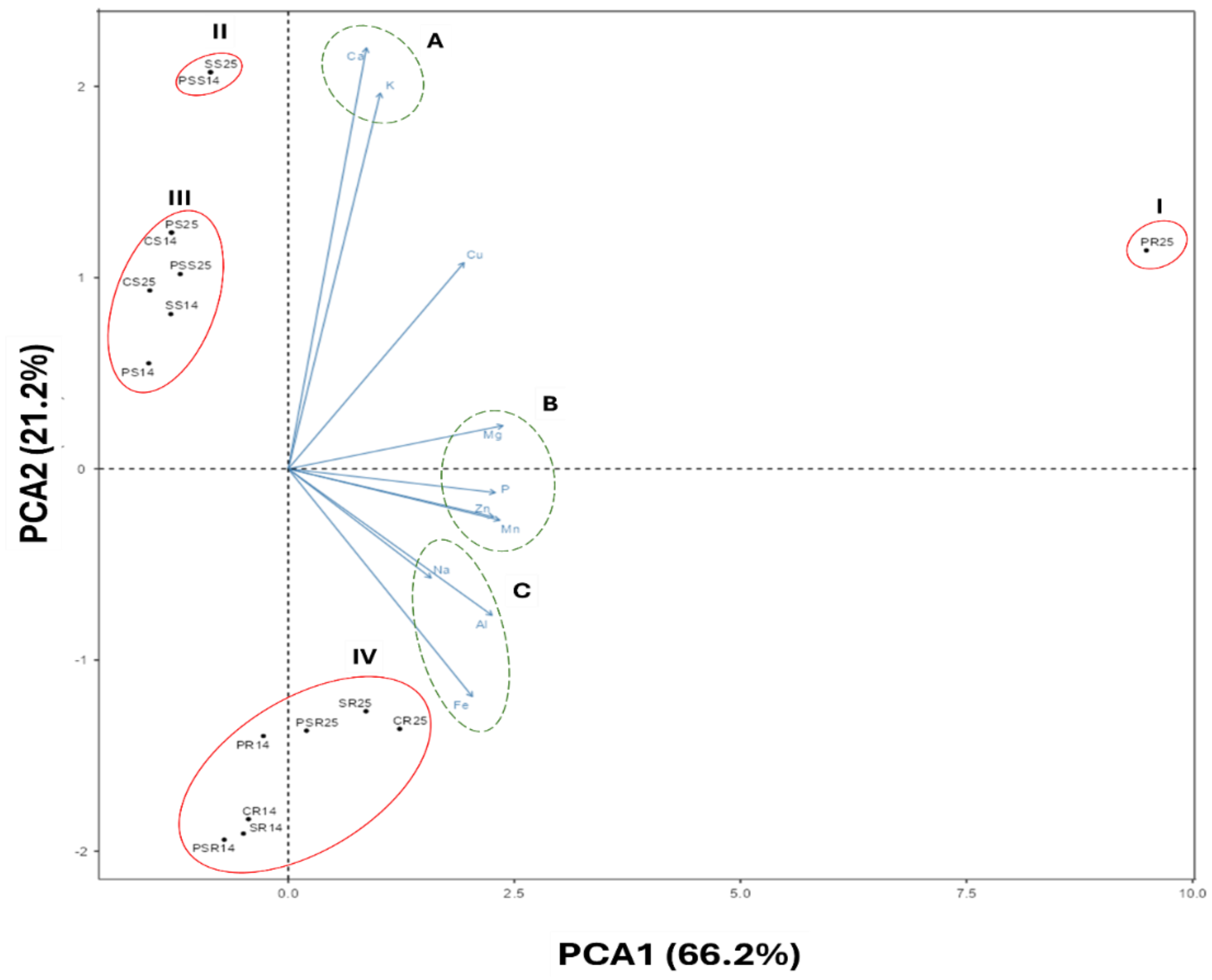

Figure 1S. Biplot (PCA1 = 66.2%, PCA2 = 21.2%) displaying nutrient loadings and the separation of treatments based on multivariate nutrient variation. Where CS14 / CS25 = Control shoot (BGN-14 / BGN-25), PS14/ PS25 = Primed shoot (BGN-14 / BGN-25), SS14 / SS25 = Salt-stressed shoot (BGN-14 / BGN-25), PSS14 / PSS25 = Primed + salt shoot (BGN-14 / BGN-25), CR14 / CR25 = Control root (BGN-14 / BGN-25), PR14 / PR25 = Primed root (BGN-14 / BGN-25), SR14 / SR25 = Salt-stressed root (BGN-14 / BGN-25), PSR14 / PSR25 = Primed + salt root (BGN-14 / BGN-25). Nutrients include; Ca = calcium, Mg= magnesium, K= potassium, P= phosphorus, Na= sodium, Zn= zinc, Cu= copper, Mn= manganese, Fe= iron, and Al= aluminium.

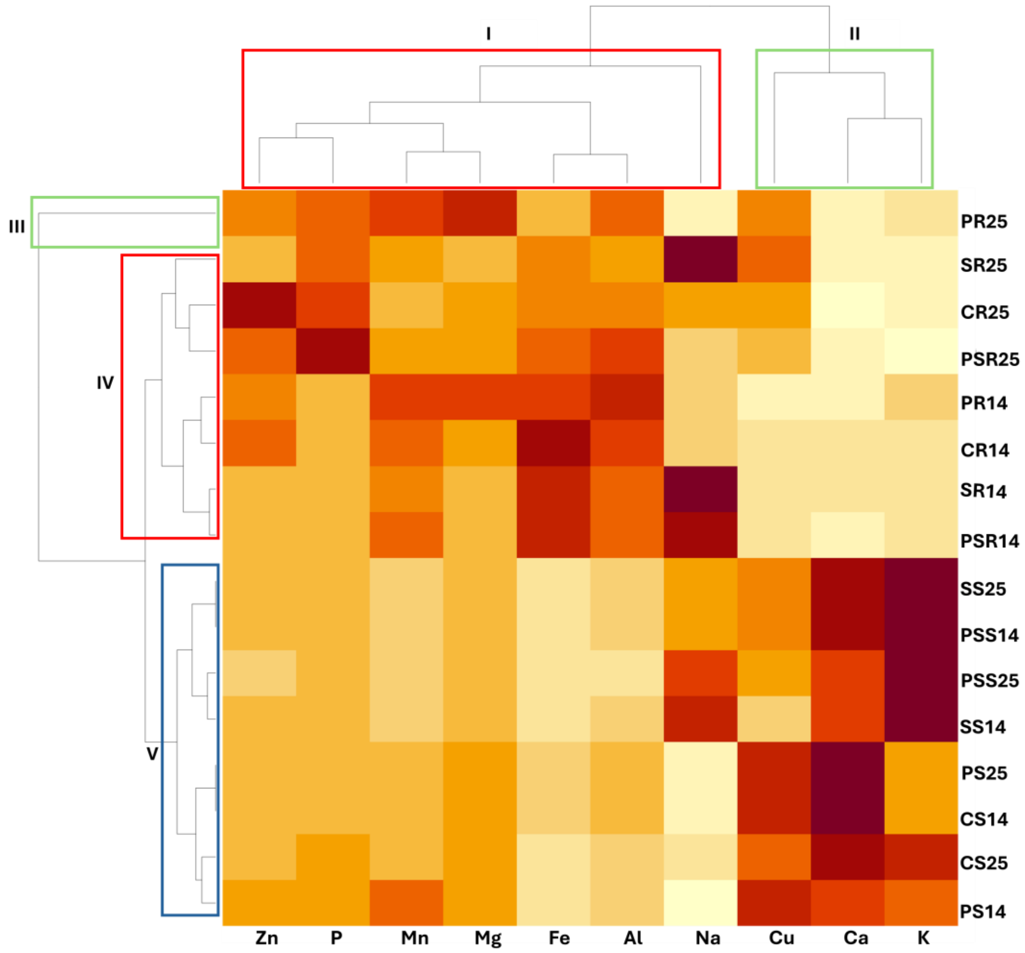

Figure 2S. Heatmap illustrating the intensity patterns of nutrient concentrations and the hierarchical clustering of similarity-based grouping of mineral profiles across treatments, tissue types, and genotypes. Where CS14 / CS25 = Control shoot (BGN-14 / BGN-25), PS14 / PS25 = Primed shoot (BGN-14 / BGN-25), SS14 / SS25 = Salt-stressed shoot (BGN-14 / BGN-25), PSS14 / PSS25 = Primed + salt shoot (BGN-14 / BGN-25), CR14 / CR25 = Control root (BGN-14 / BGN-25), PR14 / PR25 = Primed root (BGN-14 / BGN-25), SR14 / SR25 = Salt-stressed root (BGN-14 / BGN-25), PSR14 / PSR25 = Primed + salt root (BGN-14 / BGN-25). Nutrients include; Ca= calcium, Mg= magnesium, K= potassium, P= phosphorus, Na= sodium, Zn= zinc, Cu= copper, Mn= manganese, Fe= iron, and Al= aluminium.
